# The *Macleaya cordata* Symbiont: Revealing the Effects of Plant Niches and Alkaloids on the Bacterial Community

**DOI:** 10.3389/fmicb.2021.681210

**Published:** 2021-06-09

**Authors:** Fangying Lei, Xueduan Liu, Haonan Huang, Shaodong Fu, Kai Zou, Shuangfei Zhang, Li Zhou, Jianguo Zeng, Hongwei Liu, Luhua Jiang, Bo Miao, Yili Liang

**Affiliations:** ^1^School of Minerals Processing and Bioengineering, Central South University, Changsha, China; ^2^Key Laboratory of Biometallurgy, Ministry of Education, Changsha, China; ^3^Hunan Key Laboratory of Traditional Chinese Veterinary Medicine, Hunan Agricultural University, Changsha, China

**Keywords:** *Macleaya cordata*, 16S rRNA, bacterial community structure, niche differentiation, alkaloids

## Abstract

Endophytes are highly associated with plant growth and health. Exploring the variation of bacterial communities in different plant niches is essential for understanding microbe-plant interactions. In this study, high-throughput gene sequencing was used to analyze the composition and abundance of bacteria from the rhizospheric soil and different parts of the *Macleaya cordata*. The results indicated that the bacterial community structure varied widely among compartments. Bacterial diversity was observed to be the highest in the rhizospheric soil and the lowest in fruits. Proteobacteria, Actinobacteria, and Bacteroidetes were found as the dominant phyla. The genera *Sphingomonas* (∼47.77%) and *Methylobacterium* (∼45.25%) dominated in fruits and leaves, respectively. High-performance liquid chromatography (HPLC) was employed to measure the alkaloid content of different plant parts. Significant correlations were observed between endophytic bacteria and alkaloids. Especially, *Sphingomonas* showed a significant positive correlation with sanguinarine and chelerythrine. All four alkaloids were negatively correlated with the microbiota of stems. The predicted result of PICRUST2 revealed that the synthesis of plant alkaloids might lead to a higher abundance of endophytic microorganisms with genes related to alkaloid synthesis, further demonstrated the correlation between bacterial communities and alkaloids. This study provided the first insight into the bacterial community composition in different parts of *Macleaya cordata* and the correlation between the endophytic bacteria and alkaloids.

## Introduction

Endophytes are widely present inside plants during part or all stages of their life cycle. They are able to survive in the root, stem, leaf, fruit, flower, and seed due to the specific colonization conditions provided by plant tissues and organs (Loaces et al., 2010; Bulgarelli et al., 2013; Wellner et al., 2013; Glassner et al., 2015). Plant microbial community composition is influenced by environmental factors, including climate, temperature, geographic location, vegetation density, and host genotype (Overbeek and Elsas, 2008; Meyer and Leveau, 2012; Ramond et al., 2013; Campisano et al., 2017; Yana et al., 2017; Ting et al., 2019; Sharaby et al., 2020). However, several studies have shown that plant compartment is the main driver of bacterial community composition, while the season, location, and plant species only play a minor role (Miller and Rudgers, 2014; Junker and Keller, 2015; Fonseca-García et al., 2016; Cregger et al., 2018). Soil microorganisms were partially the source of endophytic bacteria (Zarraonaindia et al., 2015; Mangeot-Peter et al., 2020). They migrated and colonized the area around the root of plants under the influence of the roots’ secretions (Tahtamouni et al., 2015). Rhizospheric microbial community composition was significantly influenced by the physical and chemical parameters in soil (Winston et al., 2017; Hartman et al., 2018; Ziyuan et al., 2020), which also affected the plant compartments in part (Mighell et al., 2019). However, Two studies on Arabidopsis showed that the endophytic microbial community composition in the Arabidopsis plants growing on four different soils was similar (Davide et al., 2012; Lundberg et al., 2012), indicating the existence of host mediated control mechanisms. Significant plant compartment effects were also observed in the microbiome of plants such as *Populus*, *Cycas panzhihuaensis*, and *Stellera chamaejasme L* (Jin et al., 2014; Cregger et al., 2018; Zheng and Gong, 2019).

Endophytes and plants can be considered as mutualistic symbiosis. They survived and evolved together (Hardoim et al., 2015; Vandenkoornhuyse et al., 2015). Endophytes are an essential part of the plant micro-ecosystem. They were thought to complement the host plant’s gene library and could regulate metabolism, enhance stress resistance, and transform host plants’ secondary metabolites (Zhou et al., 2015; Prasad et al., 2018; Cordovez et al., 2019; Víctor and Juan, 2019). Some endophytic bacteria were able to synthesize bioactive compounds such as saponins, terpenoids, and alkaloids, which are potential sources of antibacterial, anti-insect, anticancer, and other properties (Yu et al., 2010; Pimentel et al., 2011; Gutierrez et al., 2012). Considering the various effects of plant-associated bacteria on plants, recording the spatial variability of these bacterial communities is critical for further understanding of plant-microbe interactions and the potential value of endophytic bacteria.

*Macleaya cordata*, a perennial plant mainly distributes in China, Europe, and North America, has been considered as a traditional folk herbal medicine. Its chemical composition and biological activity were a matter of concern because of its detoxifying, analgesic, anti-inflammatory, antimicrobial, and antitumoral properties (Liu et al., 2013; Khadem et al., 2014; Li et al., 2017; Huang et al., 2018). Its extract has been used as a good alternative of antibiotics in feed additives for animal production, and has achieved European Food Safety Certification for its effectiveness in treating inflammation and regulating the intestinal flora of livestock and poultry. Modern pharmacological studies showed that isoquinoline alkaloids are the primary bioactive substances in *Macleaya cordata*. Some of them possess a prominent apoptotic effect on cancer cells. For instance, sanguinarine could inhibit cell invasion and the MMP-9 and COX-2 expression in TPA-induced breast cancer cells by inducing HO-1 expression (Park et al., 2014). The four main alkaloids in *Macleaya cordata* are sanguinarine, chelerythrine, allocrytopine, and protopine (Kosina et al., 2010). Significant differences in the accumulation of these four alkaloids were reported in the root, stem, leaf, and fruit of *Macleaya cordata*. Sanguinarine and chelerythrine were observed to accumulate mainly in the fruits, with allocrytopine and protopine being most abundant in roots and leaves, respectively. The alkaloids content in the stem was very low. Endophytic bacterial communities in different parts might be affected by this difference.

Bacterial communities across different niches covering below-ground and above-ground tissue-level microbial habitats (rhizospheric soil, root, stem, leaf, and fruit) were characterized in this study by using 16S rRNA gene-targeted Illumina MiSeq sequencing. The content of four main alkaloids was also detected to explore the relationship between alkaloids and endophytic bacteria. We hypothesized that: (i) Niche differentiation among different sample types influenced the composition and diversity of the *Macleaya cordata* associated microbial communities (ii) Alkaloids might contribute to the variation of bacterial communities in different niches. This experiment can support the subsequent search of functional microorganisms and guide the cultivation, protection, and increase of crucial metabolites and resource utilization of *Macleaya cordata*.

## Materials and Methods

### Study Location and Sampling Methods

*Macleaya cordata* used in this study were collected from an experimental cultivar trial in Hunan province at a site managed by the Hunan Agricultural University in July 2019. Mature and healthy roots, stems, leaves, fruits, and rhizospheric soil were selected from six individual plants on the experimental blocks. All six trees were growing in the same general area and experienced similar developing conditions. Samples were collected randomly and cleaned with distilled water, followed by cutting three pieces from each leaf (from the bottom, middle, and top) with five leaves from different branches. Roots, stems, and fruits were also collected from the same location from each tree (Roots and stems were cut into 2.5 cm long pieces using a sterilized scalpel). All samples were collected aseptically wearing bioclean gloves and put in marked aseptic bags. They were placed in dry ice foam boxes and taken back to the lab as soon as possible. The part for alkaloid content detection was frozen in liquid nitrogen immediately and stored in a −80°C freezer. The part for microbiological analysis would be surface sterilized firstly. Then samples were crushed and homogenized to powder using a sterilized mortar and pestle, frozen in liquid nitrogen, and stored at −80°C until DNA extraction. The surface disinfection process is as follows: The samples were rinsed in tap water, sterilized by 75% ethanol (1 min), washed with sterile water three times, soaked in 8% sodium hypochlorite (5 min), washed with sterilized water five times, and left to dry with sterile filter paper.

### Preparation of Standard Alkaloid Solutions and Sample Solutions

All solvents used were of liquid chromatography (LC) grade. The sanguinarine and chelerythrine standards were purchased from Sigma (Shanghai, China), while protopine and allocryptopine standards were purchased from the National Institute for the Control of Pharmaceutical and Biological Products (Beijing, China). Standards were dissolved in methanol and diluted to obtain a series of working solutions (5.12–409.52, 4.20–84.04, 8.61–550.75, 7.80–499.30 μg/ml, respectively) and establish the standard curves at 280 nm. 5.0 g of the powder (60 mesh) of each sample was placed in 250 ml corked conical flasks and extracted with 100 ml of methanol-1% HCL (50:50) in an ultrasonic bath (60 min, 35°C, 200 W, 40 KHz), the weight loss was compensated with methanol-1% HCL, and the supernatant was collected. The supernatant was filtered through a 0.22 μm membrane before HPLC analysis. Then a 20 μl aliquot of the solution was injected into the HPLC system. For accurate analysis of high content alkaloids, the sample solution was diluted to avoid a concentration that lies outside the linear range.

### HPLC Analysis

Analysis was carried out using a Waters alliance 1,260 liquid chromatographic system (Milford, MA, United States). LC separations were accomplished on a 5 μm, 250 mm × 4.6 mm Ultimate XB C18 column (Welch Materials, Ellicott City, United States) at 35°C. Mobile phases consisted of (A) water containing Acetonitrile and (B) 1% phosphoric acid solution. Gradient elution program was as follows: 0–11 min 25% A, 11–27 min 25–60% A, 27–29 min 60–25% A, 29–35 min 25% A. The flow rate was set at 1 ml/min. The injection volume was 20 μl. A wavelength of 280 nm was selected for quantification. The limit of detection (LOD) was 1.35, 0.97, 0.89, 0.93 μg/ml for protopine, allocryptopine, sanguinarine, and chelerythrine.

### DNA Extraction and PCR Amplification of the Bacterial 16S rRNA

Total plant DNA and soil microbial DNA were extracted using DNeasy^®^ Plant Mini Kit (250) (Qiagen, Inc., Duesseldorf, Germany) and ALFA-SEQ Advanced Soil DNA Kit (Guangdong Magigene Biotechnology Co., Ltd.), respectively. All steps were performed strictly according to the manufacturer’s protocols. The concentration and purity of DNA were determined using a NanoDrop ND-1000 spectrophotometer (NanoDrop Technologies, Wilmington, United States). 1% (w/v) TAE-agarose gel stained with EB was also used to detect the extracted DNA’s quality. The V5 and V6 hypervariable regions of the bacteria 16S rRNA genes were amplified with the primers 799F (5′-AAC MGG ATT AGA TAC CCK G-3′); 1115R (5′-AGG GTT GCG CTC GTT G-3′). The extracted DNA was amplified using a thermocycler polymerase chain reaction (PCR) system with the following process: denaturation for 1 min at 94°C; 35 cycles of 20 s at 94°C, annealing for 25 s at 57°C, elongation for 45 s at 68°C; and a final extension step for 10 min at 68°C. The PCR was performed with reaction volumes of 50 μl containing 25 μl 2 × Taq Master Mix plus (Vazyme), 1.5 μl each primer (10 μM), 2 μl template DNA (100 ng), 20 μl PCR water. The PCR products were assessed by 1.5% (w/v) TAE-agarose gel stained with EB and further purified using the Gel Extraction Kit D2500 (OMEGA bio-tek, Norcross, Georgia, United States). High-throughput paired-end sequencing of the purified amplicons was conducted on the Illumina Miseq platform (Miseq PE250).

### Sequencing Data Processing

Gene sequencing data of raw 16S rRNA were imported to the QIIME2 platform to generate the OTU table. The cutadapt program was used to remove the forward and reverse primers. The paired-end fastq files were merged, denoised, chimeras removed using the dada2 program. Meanwhile, they were clustered to operational taxonomic units (OTUs) at 97% similarity. Clean data were classified using BLAST with the Greengenes reference databases. Contaminant sequences unclassified at the domain (bacteria/archaea), mitochondria, and chloroplasts were filtered during the taxa filter-table process. All sequences produced from Illumina sequencing had been uploaded to the sequence read archive (SRA) of the NCBI database. The accession number of all samples is PRJNA714300.

### Statistical Analysis

Alpha-diversity indexes, including Shannon index and Pielou evenness, and Non-metric Multidimensional Scaling (NMDS) for assessing the beta-diversity differences in community composition were calculated on Institute for Galaxy | Denglab pipeline^1^. Dissimilarity tests of OTUs and predicted function genes (by PICRUSt2) were computed using MRPP, ANOSIM, and PERMANOVA with Bray-Curtis distance matrices to identify whether the habitat had a significant effect on community composition and the abundance of functional genes. Statistical analyses were conducted using Minitab 19.0 software (Minitab Inc., Pennsylvania State University, Pennsylvania, United States). One-way ANOVA analyses, followed by Tukey’s test, were carried out to identify the gene abundance difference. Correlation between bacterial community and four alkaloids was carried out based on the Spearman correlation coefficients and Canonical Correspondence Analysis (CCA). PICRUSt2 was employed to predict each sample’s function genes based on the 16S rRNA sequencing data, and the KEGG database was used to match the selected reference OTU.

## Results

### Analysis of Sequencing Data

A total of 4,298,014 raw reads were obtained among 30 samples. After quality control and contaminant sequences removal, 1,218,950 high-quality paired 16S rRNA sequences with an average read length of 300 bp remained, which can be clustered into 6,866 OTUs (Supplementary Table 1). CPM standardization was performed on all samples before analysis of microbial communities. Rarefaction curves were constructed for each sample showing the number of observed OTUs. As expected, endophytic microorganisms had lower observed OTUs than rhizospheric soil bacterial communities (Supplementary Figure 1). Rarefaction curves evaluating the OTU richness per sample generally approached saturation, indicating that the sequencing depth was adequate.

### Diversity of the Microbial Communities Associated With *Macleaya cordata*

**FIGURE 1:**
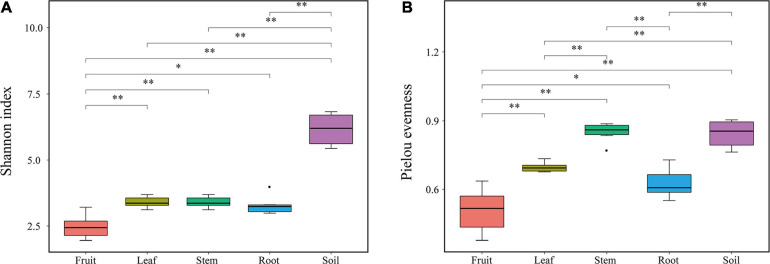
Diversity indexes of endophytic bacterial communities. **(A)** Shannon index **(B)** Pielou’s evenness.

**FIGURE 2:**
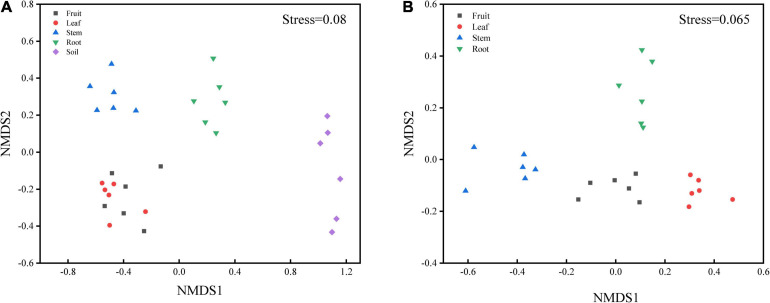
Non-metric multidimensional scaling (NMDS) carried out with **(A)** OTU table based on Bray-Curtis distance (S = 0.08) and **(B)** predicted function gene table based on Bray-Curtis distance (S = 0.065).

Bacterial diversity was observed to be highest in soil, while the situation in plants was relatively complex, with bacterial diversity showing no significant differences in roots, stems and leaves, but a significant decrease in fruits. The highest evenness of bacterial communities was found in the stems among plant parts (Figure 1). NMDS revealed strong clustering of bacterial communities based on plant compartments (Figure 2A). Specifically, Coordinate1 (x-axis) separated the rhizospheric soil, roots, and above-ground (stems, leaves, fruit) samples. Rhizospheric soil exhibited a significant difference in community composition comparing with the other four plant compartments. However, the bacterial communities in the fruit and leaf were highly similar and separated from other endosphere samples in Coordinate2 (y-axis). Dissimilarity tests based on Bray-Curtis distance, including MRPP, ANOSIM, and PERMANOVA, also showed that the bacterial community composition of these five groups was significantly different (*P* < 0.05) (Table 1).

**TABLE 1:** Dissimilarity tests of OTUs and predicted function genes (by PICRUSt) based on Bray-Curtis distance.

	MRPP		ANOSIM		PERMANOVA	
	r	p	r	p	r	p
OTUs	0.6037	**0.001**	0.9042	**0.001**	8.3007	**0.001**
function genes	0.3827	**0.001**	0.5728	**0.001**	10.5800	**0.001**

*Significant differences (*P* < 0.05) are indicated in bold.*

To better understand the OTUs distribution within different niches, the number of OTUs shared by all compartments and OTUs uniquely identified in each sample type were calculated (Supplementary Figure 2). The bacterial communities in the rhizospheric soil had the highest number of OTUs, followed by roots. 302, 155, 236, 245, and 5,391 bacterial OTUs were uniquely identified for root, stem, leaf, fruit, and rhizospheric soil, respectively, with 19 OTUs shared among the five sample groups. A high overlap (208) of OTUs from rhizospheric soil and root was observed.

### The Bacterial Composition Differences Across Habitat at Phylum Level and Genera Level

**FIGURE 3:**
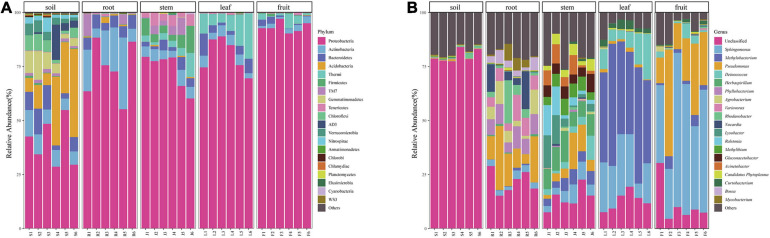
Bacterial community composition of different niches **(A)** bacterial community at the Phylum level and **(B)** bacterial community at the genera level.

At the phylum level, the bacterial community composition showed high variabilities among different compartments (Figure 3A). Twenty-five dominant bacterial phyla (≥0.1% relative abundance) were detected across this study. Proteobacteria phyla was the dominant group in all niches (soil, root, stem, leaf, fruit), representing 28.61–54.84%, 37.76–88.36%, 60.43–79.27%, 69.53–88.93%, and 90.30–97.10% of all bacteria, respectively. Alphaproteobacteria accounted for the highest proportion in leaves. The phylum Thermi showed a significantly high abundance in the above-ground part, almost absent in the underground part. Firmicutes and Tenericutes were detected in stems but rare in other parts. The highest abundance of Actinobacteria was found in the roots, followed by the rhizospheric soil, with lower abundance in the above-ground parts. Bacteroidetes had greater abundance in soils and roots than in the above-ground parts whereas TM7 had a greater abundance in root and stem habitats than other habitats. Bacterial community composition was different between rhizospheric soil and plant compartments. Acidobacteria, Bacteroidetes, Gemmatimonadetes, Chloroflexi, Verrucomicrobia, AD3, and Nitrospirae were relatively abundant in the soil compared to other compartments. However, some of them were also detected in endosphere samples in very low abundance.

At the genera level, 334 bacteria were identified from all sequences. Dominant bacteria differed among five compartments. Only the top 20 genera were shown in Figure 3B. *Sphingomonas* (47.77%) and *Pseudomonas* (25.09%) were abundant in the fruit, whereas *Methylibium* (45.25%) and *Deinococcus* (10.20%) were mainly distributed in the leaves. *Pseudomonas* (10.09%), *Nocardia* (11.42%), and *Rhodanobacter* (8.82%) were the dominant genera in the roots, and their abundance did not differ much. The microorganisms in stems were relatively uniform. The dominant genera in fruit, leaves, and roots were also abundant in stems. Besides, some microorganisms with low abundance in other endophytic tissues like *Herbaspirillum* (9.79%), *Lysobacter* (6.30%), and *Ralstonia* (6.03%) were also present in relatively high abundance in stems. Most bacterial genera detected in soil could not be identified.

### Alkaloids Content of *Macleaya cordata*

**FIGURE 4:**
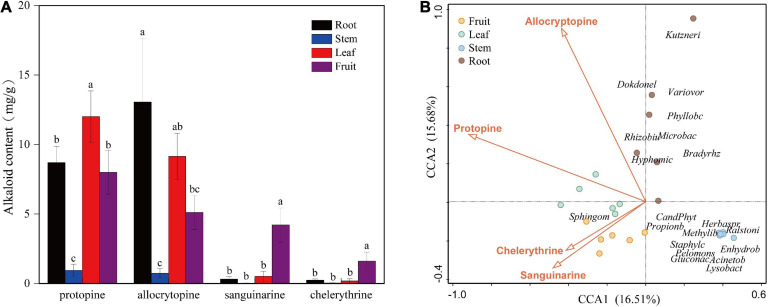
**(A)** Contents of four kinds of alkaloids in different organs (fruit, leaf, stem, and root) of *Macleaya cordata*. **(B)** Canonical Correspondence Analysis (CCA) of the top 20 bacterial genera (with significant correlation coefficients) and four critical alkaloids in *Macleaya cordata*.

The contents of four kinds of alkaloids in each tissue (fruit, leaf, stem, and root) of the six mature plants were calculated according to the standard curve regression equation (Supplementary Table 2). Alkaloid content showed a significant difference among organs (Figure 4A). The highest content of sanguinarine and chelerythrine was observed in fruit (4.04 and 1.65 mg/g). They were low in roots and leaves and almost zero in the stem. The accumulation of alkaloids in leaves and roots was mainly composed of protopine and allocrytopine. Roots contained the highest allocrytopine (11.67 mg/g) than other organs, while the leaf’s protopine content (12.81 mg/g) was the highest. The accumulation of four kinds of alkaloids in the stem was much lower than that in the other three organs.

### Correlation Between the Endophytic Bacterial Abundances and Four Alkaloids

The Spearman correlation analysis was performed to explore the relationship between the endophytic bacteria at the phylum and genus level (relative abundance > 0.01%) and alkaloids to quantify the environmental influence (Tables 2, 3). Tenericutes showed a significant (*P* < 0.05) and negative correlation with all four alkaloids. Actinobacteria was significantly (*P* < 0.05) negatively correlated with sanguinarine and chelerythrine. However, most of the genera with higher abundance in this phylum showed a significant (*P* < 0.05) and positive correlation with allocryptopine. The Firmicutes, β- proteobacteria, and γ-proteobacteria were significantly (*P* < 0.05) negatively correlated with protopine and allocryptopine. Only the α-proteobacteria was significantly (*P* < 0.05) and positively correlated with protopine, allocryptopine, and sanguinarine. At the genera level, most endophytic bacteria were significantly (*P* < 0.05) correlated with alkaloids. *Sphingomonas* showed a significant (*P* < 0.05) positive correlation with sanguinarine and chelerythrine, while *Herbaspirillum*, *Methylibium*, and *Pelomonas* showed a significant (*P* < 0.01) and negative correlation with them. Canonical Correspondence Analysis (CCA) of the top 20 bacterial genera (with significant correlation coefficients) and four critical alkaloids in *Macleaya cordata* was also conducted (Figure 4B). The results were consistent with the above analysis. The sanguinarine and chelerythrine were more associated with the fruit bacterial communities, whereas protopine and allocryptopine had greater effects on leaf and root bacterial communities, respectively. However, all four alkaloids were negatively correlated with the microbiota of the stem.

**TABLE 2:** Spearman Correlation coefficients showing the relationships between endophytic bacterial phyla and four alkaloids.

Phyla	Protopine		Allocryptopine		Sanguinarine		Chelerythrine	
	*r*	*P*	*R*	*p*	*R*	*p*	*r*	*p*
Acidobacteria	−0.243	0.253	0.171	0.423	−0.241	0.257	−0.151	0.482
Actinobacteria	−0.036	0.867	0.337	0.108	−0.531	**0.008**	−0.424	**0.039**
AD3	0.144	0.503	0.411	**0.046**	0.067	0.756	0.080	0.710
Armatimonadetes	0.163	0.446	0.192	0.368	0.089	0.681	0.145	0.500
Bacteroidetes	0.137	0.525	0.378	0.069	−0.316	0.133	−0.332	0.113
Chlamydiae	−0.010	0.962	0.293	0.165	−0.281	0.184	−0.266	0.209
Chlorobi	0.122	0.571	0.607	**0.002**	−0.151	0.480	0.077	0.722
Chloroflexi	0.348	0.096	0.621	**0.001**	0.198	0.354	0.296	0.160
Cyanobacteria	−0.256	0.227	−0.256	0.227	−0.288	0.173	−0.302	0.152
Euryarchaeota	−0.095	0.658	0.051	0.814	−0.187	0.381	−0.114	0.595
FBP	0.227	0.286	0.437	**0.033**	−0.104	0.628	−0.103	0.632
Firmicutes	−0.763	**0.000**	−0.713	**0.000**	−0.370	0.075	−0.267	0.207
Fusobacteria	0.219	0.303	0.263	0.214	0.126	0.557	0.144	0.502
Gemmatimonadetes	−0.035	0.872	0.180	0.401	0.031	0.886	0.050	0.815
Nitrospirae	0.105	0.624	0.256	0.227	0.030	0.888	0.106	0.624
OD1	0.151	0.482	0.316	0.132	−0.151	0.480	−0.121	0.575
Planctomycetes	−0.111	0.605	0.049	0.820	−0.321	0.126	−0.265	0.211
Proteobacteria	0.051	0.813	−0.225	0.289	0.608	**0.002**	0.577	**0.003**
SR1	0.286	0.175	0.226	0.288	0.166	0.437	0.166	0.439
Tenericutes	−0.481	**0.017**	−0.544	**0.007**	−0.685	**0.000**	−0.659	**0.000**
Thermi	0.283	0.180	−0.173	0.419	0.000	0.998	−0.231	0.277
TM6	−0.083	0.699	0.302	0.151	0.178	0.404	0.321	0.126
TM7	−0.129	0.548	−0.002	0.995	−0.495	**0.014**	−0.418	**0.042**
Verrucomicrobia	−0.035	0.871	0.296	0.160	−0.251	0.237	−0.090	0.675
WPS-2	0.151	0.482	0.316	0.132	−0.151	0.480	−0.121	0.575

*Significant differences (*P* < 0.05) are indicated in bold.*

**TABLE 3:** Spearman Correlation coefficients showing the relationships between endophytic bacterial genera (relative abundance > 0.01%) and four alkaloids.

Phylum	Genus	Protopine		Allocryptopine		Sanguinarine		Chelerythrine	
		*r*	*P*	*r*	*p*	*r*	*p*	*r*	*p*
Thermi		0.283	0.180	−0.173	0.419	**0.000**	0.998	−0.231	0.277
	*Deinococcus*	0.283	0.181	−0.170	0.426	0.012	0.955	−0.219	0.304
Actinobacteria		−0.036	0.867	0.337	0.108	−0.531	**0.008**	−0.424	**0.039**
	*Amycolatopsis*	0.100	0.642	0.319	0.129	−0.123	0.567	0.010	0.964
	*Curtobacterium*	0.444	**0.030**	0.004	0.985	0.228	0.284	0.047	0.828
	*Kineococcus*	0.658	**0.000**	0.262	0.216	0.350	0.094	0.157	0.463
	*Kribbella*	0.102	0.634	0.493	**0.014**	−0.003	0.987	0.110	0.608
	*Kutzneria*	0.034	0.876	0.467	**0.022**	−0.032	0.882	0.164	0.445
	*Microbacterium*	0.387	0.062	0.726	**0.000**	0.125	0.560	0.207	0.332
	*Mycobacterium*	−0.070	0.745	0.405	**0.050**	−0.128	0.550	0.025	0.908
	*Nocardia*	0.140	0.515	0.451	**0.027**	0.013	0.951	0.107	0.620
	*Propionibacterium*	−0.487	**0. 016**	−0.628	**0.001**	−0.067	0.755	−0.058	0.788
	*Pseudonocardia*	0.326	0.119	0.491	**0.015**	0.194	0.365	0.204	0.338
	*Streptomyces*	0.344	0.100	0.496	**0.014**	0.117	0.586	0.238	0.262
Bacteroidetes		−0.341	0.103	−0.457	**0.025**	−0.302	0.152	−0.319	0.129
	*Dyadobacter*	−0.129	0.548	0.339	0.105	−0.008	0.972	0.193	0.365
	*Hymenobacter*	0.527	**0.008**	0.088	0.684	0.269	0.204	0.081	0.707
	*Spirosoma*	0.643	**0.001**	0.236	0.268	0.442	**0.031**	0.235	0.269
	*Chryseobacterium*	−0.054	0.803	−0.176	0.411	0.176	0.409	0.125	0.562
	*Pedobacter*	−0.312	0.138	−0.005	0.982	0.109	0.613	0.247	0.245
Firmicutes		−0.763	**0.000**	−0.713	**0.000**	−0.37	0.075	−0.267	0.207
	*Bacillus*	−0.257	0.226	0.081	0.705	−0.469	**0.021**	−0.270	0.202
	*Staphylococcus*	−0.728	**0.000**	−0.701	**0.000**	−0.328	0.118	−0.270	0.203
α-Proteobacteria		0.804	**0.000**	0.465	**0.023**	0.501	**0.013**	0.368	0.077
	*Bradyrhizobium*	−0.388	0.061	0.151	0.480	−0.455	**0.026**	−0.220	0.303
	*Methylobacterium*	0.263	0.215	−0.203	0.339	−0.121	0.572	−0.316	0.132
	*Afipia*	0.087	0.687	0.614	**0.001**	−0.115	0.592	0.096	0.654
	*Agrobacterium*	−0.035	0.872	0.261	0.218	−0.103	0.633	0.030	0.891
	*Bosea*	0.039	0.856	0.317	0.131	0.203	0.340	0.320	0.127
	*Caulobacter*	−0.253	0.232	0.038	0.860	−0.096	0.656	0.015	0.946
	*Hyphomicrobium*	−0.230	0.279	0.220	0.302	−0.318	0.129	−0.116	0.589
	*Novosphingobium*	−0.128	0.551	0.159	0.459	0.088	0.682	0.231	0.276
	*Phyllobacterium*	−0.145	0.499	0.329	0.117	−0.093	0.666	0.098	0.648
	*Rhizobium*	−0.132	0.539	0.364	0.080	−0.173	0.419	0.056	0.797
	*Rhodoplanes*	0.077	0.721	0.522	**0.009**	−0.073	0.733	0.052	0.809
	*Sphingomonas*	0.257	0.226	−0.196	0.358	0.554	**0.005**	0.428	**0.037**
β-Proteobacteria		−0.718	**0.000**	−0.570	**0.004**	−0.386	0.063	−0.249	0.242
	*Delftia*	−0.089	0.681	0.275	0.193	0.245	0.249	0.382	0.066
	*Herbaspirillum*	−0.660	**0.000**	−0.448	**0.028**	−0.751	**0.000**	−0.675	**0.000**
	*Janthinobacterium*	0.564	**0.004**	0.169	0.429	0.495	**0.014**	0.379	0.068
	*Methylibium*	−0.726	**0.000**	−0.525	**0.008**	−0.760	**0.000**	−0.649	**0.001**
	*Pelomonas*	−0.781	**0.000**	−0.691	**0.000**	−0.553	**0.005**	−0.509	**0.011**
	*Polaromonas*	−0.070	0.744	0.244	0.250	−0.122	0.570	0.014	0.947
	*Ralstonia*	−0.815	**0.000**	−0.775	**0.000**	−0.318	0.130	−0.264	0.213
	*Variovorax*	0.105	0.626	0.552	**0.005**	−0.031	0.886	0.098	0.650
γ-Proteobacteria		−0.755	**0.000**	−0.564	**0.005**	−0.068	0.753	0.064	0.766
	*Acinetobacter*	−0.867	**0.000**	−0.707	**0.000**	−0.395	0.056	−0.305	0.147
	*Dokdonella*	0.102	0.635	0.641	**0.001**	−0.117	0.585	0.097	0.652
	*Enhydrobacter*	−0.856	**0.000**	−0.824	**0.000**	−0.324	0.123	−0.233	0.273
	*Erwinia*	−0.227	0.285	−0.334	0.111	0.231	0.277	0.235	0.269
	*Gluconacetobacter*	−0.769	**0.000**	−0.715	**0.000**	−0.397	0.055	−0.397	0.055
	*Klebsiella*	−0.126	0.558	−0.257	0.225	0.023	0.914	0.012	0.955
	*Lysobacter*	−0.683	**0.000**	−0.670	**0.000**	−0.332	0.113	−0.352	0.092
	*Pantoea*	−0.053	0.805	−0.303	0.151	0.373	0.073	0.366	0.079
	*Pseudomonas*	−0.369	0.076	−0.363	0.082	0.356	0.087	0.447	**0.029**
	*Rheinheimera*	−0.434	**0.034**	−0.389	0.060	−0.415	**0.044**	−0.317	0.131
	*Rhodanobacter*	−0.251	0.238	0.297	0.158	−0.127	0.553	0.111	0.606
	*Serratia*	−0.392	0.058	−0.429	**0.036**	−0.310	0.141	−0.296	0.160
	*Stenotrophomonas*	−0.067	0.757	−0.172	0.421	0.313	0.137	0.341	0.103
	*Steroidobacter*	0.075	0.729	0.540	**0.007**	−0.021	0.923	0.189	0.377
δ-Proteobacteria		0.253	0.233	0.086	0.689	0.102	0.636	0.108	0.617
	*Cystobacter*	0.426	**0.038**	0.068	0.752	0.406	**0.049**	0.244	0.251
Tenericutes		−0.481	**0.017**	−0.544	**0.007**	−0.685	**0.000**	−0.659	**0.000**
	*Candidatus Phytoplasma*	−0.478	**0.018**	−0.541	**0.007**	−0.685	**0.000**	−0.653	**0.001**

*Significant differences (*P* < 0.05) are indicated in bold.*

### Functional Predictions by PICRUSt2

PICRUSt2 was applied as an exploratory predictive tool for functional annotation analysis. 7,185 KEGG Orthology groups (KOs) were predicted in *Macleaya cordata* endophytic communities. NMDS showed that these KOs were strongly clustered and separated from each other in different niches (Figure 2B). Dissimilarity tests of the relative abundance of KOs based on Bray-Curtis distance were also employed, including MRPP, ANOSIM, and PERMANOVA (Table 1). These results revealed that the relative abundance of KOs was significantly different among the root, stem, leaf, and fruit samples (*P* < 0.05).

**FIGURE 5:**
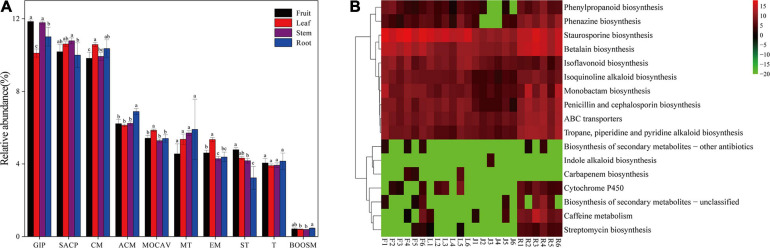
**(A)** Gene functional predictions of endophytic microbiota between different plant compartments. (GIP: genetic information processing; SACP: signaling and cellular processes; CM: Carbohydrate metabolism; ACM: Amino acid metabolism; MOCAV: Metabolism of cofactors and vitamins; MT: Membrane transport; EM: Energy metabolism; ST: Signal transduction; T: Translation; BOOSM: Biosynthesis of other secondary metabolites). **(B)** Comparisons of the seventeen gene pathways involved in the biosynthesis of secondary metabolites in the bacterial microbiota. Values of each functional gene (row) were log2 transformed. The second level of the KEGG pathway related to the biosynthesis of secondary metabolites was shown in the heatmap (S, Soil; R, Root; J, Stem; L: leaf; F: fruit).

Forty-two gene families were observed at Hierarchical Level 1 (KOs in KEGG). The majority of the KOs belonged to Genetic Information Processing, Environmental Information Processing, and Metabolism. As shown in Figure 5A, the frequencies of categories associated with membrane transport gradually increased from 4.57% in fruit to 5.91% in roots. In comparison, the frequencies of categories associated with signal transduction decreased from 4.79% in fruit to 3.23% in roots. The abundance of KOs related to the biosynthesis of other secondary metabolites was the lowest in the stem bacterial community and the highest in the root. To further identify microbiota function differences in the biosynthesis of secondary metabolites, 17 related KEGG functional categories at the second hierarchy level were analyzed (Figure 5B). In comparison between groups with different niches, eight pathways (e.g., Streptomycin biosynthesis, Caffeine metabolism, and Cytochrome P450) were significantly more abundant in the root, while the Staurosporine biosynthesis pathway was significantly more abundant in fruit.

## Discussion

### The Composition and Diversity of Bacterial Communities Differ in Niches

Bacterial community composition was significantly (*P* < 0.01) different among plant compartments (Table 1). The NMDS diagram (Figure 2A) also showed that the separation in the bacterial community structure between different plant compartments at the OTU level, indicating differences in the microbes in different niches. Although soil microorganisms were a source of plant endophytes, the migration of soil microorganisms to the root was still mediated by the host (Davide et al., 2012; Lundberg et al., 2012). For above-ground plant tissues, endophytes also derived from horizontal dispersal in the atmosphere and vertical transmission through seeds (Maignien et al., 2014; Fonseca-García et al., 2016), which resulted in bacterial differences between above-ground organs and soil. Similar results have also been reported in maize and wheat (Xiong et al., 2020), poplar (Beckers et al., 2017), *Arabidopsis* thaliana (Davide et al., 2012), and other plant species (Edwards et al., 2015; Lu et al., 2020).

As demonstrated by rarefaction curves and the alpha diversity indices, there were significant differences in species diversity between rhizospheric soil and plant compartments of *Macleaya cordata*. As an ideal habitat for various microorganisms, the bacterial diversity of rhizospheric soil was significantly higher than roots and above-ground niches. This finding is consistent with the general view of microbial colonization (Beckers et al., 2017). No significant difference was observed in the Shannon diversity of bacterial communities in roots, stems, and leaves, while the bacterial diversity in fruits was the lowest. The bacterial diversity within the plant compartments was low, probably because plant tissues were highly variable and complex. Bacterial colonization is limited by the host’s immune system, nutritional conditions, and metabolites (Compant et al., 2010). The highest evenness of bacterial communities was found in the stem among plant parts. It should be noticed that there was no significant difference in bacterial diversity between stems and roots, while many studies have determined that bacterial diversity was highest in roots (Santana et al., 2016; Wang et al., 2019). The variation of endophytic bacterial communities in different compartments was primarily driven by tissue-specific filtering mechanisms within the host (Bonito et al., 2014; Chao et al., 2020). Under the host-mediated control, only a limited number of microorganisms could maintain a symbiotic lifestyle with the host. This pressure sequentially increased from the soil to the plant compartments (Chao et al., 2020; Pasquale et al., 2020), which might be responsible for the lowest diversity of bacterial communities in fruits of *Macleaya cordata*.

### Niche Preference Exists for Bacteria of *Macleaya cordata*

We identified several prokaryotic taxa (>0.1%), including microbiota members belonging to Proteobacteria, Actinobacteria, Acidobacteria, Bacteroidetes, TM7, Firmicutes. Bacterial diversity varied among plant-associated habitats, and the dominant phylum found in each habitat was highly comparable to other plant hosts in each habitat. Many studies have reported that plants’ bacterial microbiota is generally dominated by three major phyla (Proteobacteria, Actinobacteria, and Bacteroidetes) in both above- and below-ground tissues (Tahtamouni et al., 2015; Wang et al., 2020). This is also consistent with our study. Proteobacteria was the most abundant in the above-ground compartment, while Actinobacteria was widely distributed in the below-ground. Many microorganisms were detected in rhizospheric soil but hardly found in other niches, such as Acidobacteria, Bacteroidetes, and Gemmatimonadetes, which were often discovered in the soil in other studies (Cregger et al., 2018; Wang et al., 2020). Bacteria in the stem were affected by both the above-ground parts (leaf and fruit) and the below-ground part (root) and dominated by Proteobacteria with the enrichment of Actinobacteria, Bacilli, Mollicutes, and TM7. However, some of the microorganisms did not spread from the stem to the leaf and fruit. This might be caused by nonuniform colonization of different compartments, the microbial source difference, or other environmental factors.

As shown in Figure 3B, only a few bacterial genera were dominant in fruit and leaves. *Sphingomonas* and *Pseudomonas* were found to be the dominant bacterial genera in the fruit, and *Methylobacterium*, *Sphingomonas*, and *Deinococcus* were detected as predominant groups in the endophytic communities of the *Macleaya cordata* leaf. Different studies have reported that these genera represented a substantial part of various plant species’ endophytic microbiota (Mano and Morisaki, 2008; Delmotte et al., 2009). In the previous study, *Sphingomonas* played an essential role in plant stress tolerance, plant growth promotion, and biodegradation of polycyclic aromatic hydrocarbon (Wilkes et al., 1996; Halo et al., 2015; Asaf et al., 2020). Microbial colonization is related to its ability to adapt to the host’s internal environment and its utilization of substrates. Leaves are more often exposed to the vagaries of the environment, including nutrient stress, desiccation, and ultraviolet radiation, providing a special habitat for microorganisms (Hunter et al., 2010). *Methylobacterium*, often isolated from the leaf surface and interior, could specifically colonize the plant by profiting from methanol released by the plant (Galbally and Kirstine, 2002) and has been reported to be drought and radiation-resistant (Yoshida et al., 2017; Jorge et al., 2019; Kim et al., 2019). Therefore, *Methylobacterium* were able to successfully colonize the leave extensively (Delmotte et al., 2009). *Cystobacter* was found only in the fruits and leaves, and *Erwinia* was found only in above-ground tissues. Colonization of these two genera might be due to horizontal transmission (Frank et al., 2017).

Bacterial communities of different ecological niches were assembled under the effect of environmental filtering, ecological drift, and dispersal limitations (Nemergut et al., 2013; Moroenyane et al., 2021). However, there is still a lack of understanding of these processes. Plants are exposed to diverse and highly variable environmental factors, physiological structure (thickness and shape), chemical properties (nutrients contents, water, and secondary metabolites) of each compartment drive the differences in bacterial communities to some extent (Delmotte et al., 2009; Hunter et al., 2010; Arturo et al., 2012). In a study on soybean, the secondary metabolites (ethylamine and betaine) were considered as a robust environmental filter for bacterial communities (Shintaro et al., 2019). Furthermore, the bacterial communities in *Stevia rebaudiana* and *Coptis teeta*, were also demonstrated to be significantly correlated with secondary metabolites (Yu et al., 2015; Liu et al., 2020). On the other hand, the functional capacity of bacterial species is key to their recruitment by hosts, and Burke et al. (2011) proposed that bacterial community assembly is associated with function rather than the taxonomy.

### The Alkaloids May Contribute to the Variation of the Endophytic Bacterial Communities

Protopine and allocrytopine are considered as precursors of sanguinarine and chelerythrine, respectively, and they are abundant in the root. In a previous study on the dynamics of the four alkaloids of *Macleaya cordata*, it was found that after entering the mature fruiting season, the content of protopine in the fruit decreased significantly, while the content of sanguinarine and chelerythrine increased rapidly. This suggested that protopine and allocrytopine were transported into the fruit and converted into sanguinarine and chelerythrine. The transcriptome, proteome, and metabolism data in a previous study conducted by Jianguo Zeng et al. (2013) revealed that the root of *Macleaya cordata* is the primary organ for the biosynthesis of isoquinoline alkaloids. In this study, significant differences were found in alkaloids accumulation in various organs. The content of allocrytopine in the root, leaf, and fruit showed a decreasing trend. Besides, the highest accumulation of protopine was found in leaves, followed by roots and fruit. The sanguinarine and chelerythrine in fruit were much higher than those in other tissues. The accumulation of all four alkaloids in stems was low. Some common endophytes, such as Firmicutes, have been reported to be abundant in the above-ground part, whereas in this study, it was only abundant in the stem. It can be speculated that this may explain to some extent the higher microbial diversity in stems since these four alkaloids have antibacterial effects (Beuria et al., 2005; Li et al., 2009; Razan et al., 2014; Yu et al., 2014). The CCA analysis also showed that all four alkaloids were negatively correlated with the microbiota of the stem of *Macleaya cordata*. The correlation tests between alkaloids and microorganisms also showed that most endophytic bacteria were significantly (*P* < 0.05) correlated with alkaloids.

Endophytes and host plants are closely related, and they adapt to each other and coevolve. Studies have shown that genes and abilities that evolve in one lineage are usually acquired steadily by another lineage (Papke and Gogarten, 2012). Direct gene transfer between species has occurred in all major taxa and seems to occur more frequently in prokaryotes (Moran, 2007). This leads to the possibility that microorganisms could respond to environmental toxins by selecting specific gene sequences that give them a competitive advantage over other organisms. It can be hypothesized that the microbes colonized in the plant might be affected by alkaloids produced by the host. Through the analysis of the functional annotation in Hierarchical level 2, endophytic bacteria in roots was found to contribute most to the gene abundance of the Cytochrome P450, ABC transporters, and secondary metabolite synthesis pathway including the isoquinoline alkaloid synthesis pathway. This was consistent with Jianguo Zeng’s reports that all enzymes for protopine and allocrytopine biosynthesis were highly expressed in the host root. Cytochrome P450 is considered as a large family of enzymes involved in many important metabolic pathways, and many enzymes related to the biosynthesis of benzylisoquinoline alkaloids belong to the P450 family (Zeng et al., 2013). Simultaneously, the ABC transporters are involved in transporting secondary metabolites such as alkaloids (Shitan and Yazaki, 2007). The number of genes annotated to the Staurosporine biosynthesis pathway was significantly higher in fruits than that in other parts, which may be due to the high accumulation of sanguinarine in the fruit. Staurosporine, an alkaloid with a diindole chemical structure, has been reported to partially block the accumulation of sanguinarine induced by a fungal activator (Peter et al., 1996). Since the primary function of stems is transport and there is almost no alkaloid stored in the stem, this may contribute to the low gene abundance of the microbiota genes related to the synthesis of secondary metabolites in the stem.

## Conclusion

This study provided the first insight into the bacterial communities of different plant tissues and rhizospheric soil in *Macleaya cordata*. There were significant differences in bacterial communities among different ecological niches under the influence of plants’ vertical stratification structure. A strong correlation between the endophytic bacteria and the alkaloids was found by the Spearman correlation analysis. The predicted results of PICRUST2 further demonstrated that alkaloids might contribute to the variation of bacterial communities in different niches. All in all, this experiment can support the subsequent search of functional microorganisms and guide the cultivation, protection, and increase of crucial metabolites and resource utilization of *Macleaya cordata*.

## Data Availability Statement

The datasets presented in this study can be found in online repositories. The names of the repository/repositories and accession number(s) can be found in the article/Supplementary Material.

## Author Contributions

FL, XL, and YL conceived and designed the work. FL, HH, and SF performed the experiments. FL and XL wrote and revised the paper. KZ and SZ helped with the sample collection. FL and XL contributed equally to this study. All authors read and approved the final manuscript.

## Conflict of Interest

The authors declare that the research was conducted in the absence of any commercial or financial relationships that could be construed as a potential conflict of interest.

## Footnotes

1 http://mem.rcees.ac.cn:8080/
