# Supplementary material for: The Macleaya cordata Symbiont: Revealing the Effects of Plant Niches and Alkaloids on the Bacterial Community
Source: Front Microbiol. 2021 Jun 9;12:681210. doi: 10.3389/fmicb.2021.681210 (PMC8219869; doi:10.3389/fmicb.2021.681210)

Supplementary Material

# Supplementary Figures

**Figure 1.** Rarefaction curves of Macleaya cordata plant compartment (root, stem, leaf, fruit) and rhizosphere soil.


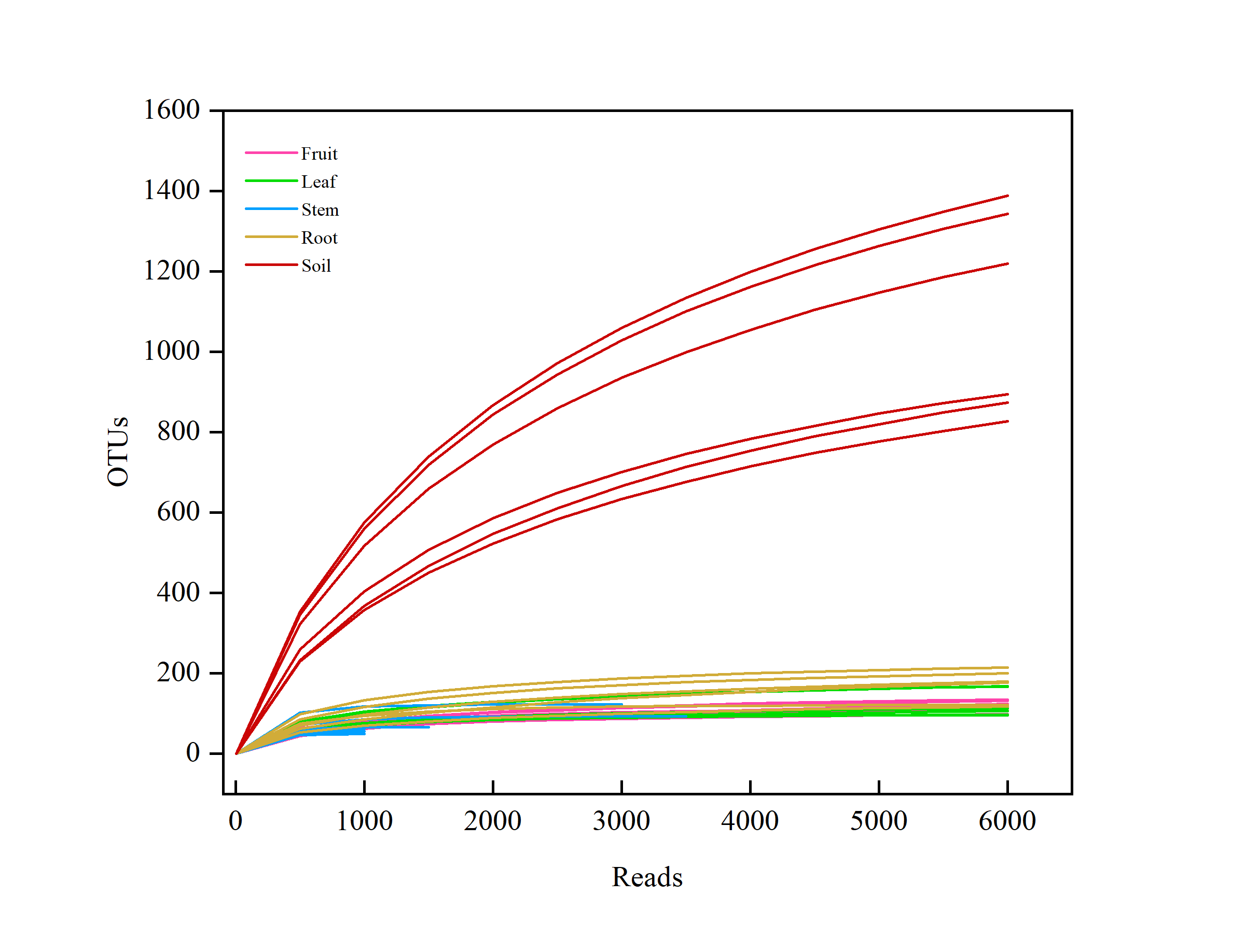


**Figure 2.** Venn diagram of samples at OTU level. The numbers in the figure represented OTUs shared or uniquely identified in different niches.


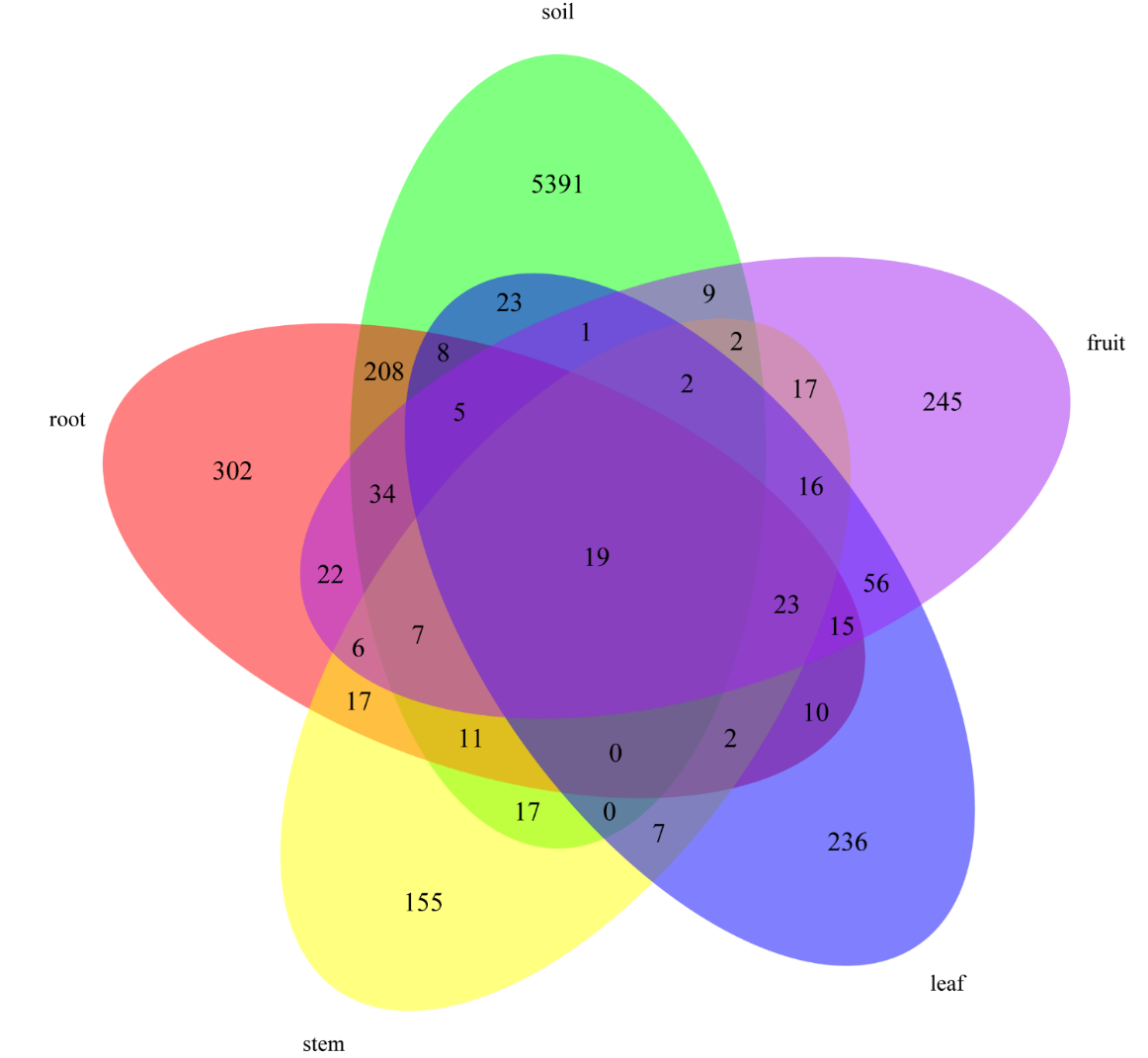

Supplement: Supplementary file 1 [file Data_Sheet_1.ZIP › supplementary materials/Supplementary_Figure.docx]
