# Supplementary material for: The Macleaya cordata Symbiont: Revealing the Effects of Plant Niches and Alkaloids on the Bacterial Community
Source: Front Microbiol. 2021 Jun 9;12:681210. doi: 10.3389/fmicb.2021.681210 (PMC8219869; doi:10.3389/fmicb.2021.681210)
Supplement: Supplementary file 1 [file Data_Sheet_1.ZIP › supplementary materials/Supplementary_Table.docx]

Supplementary Material

# Supplementary Figures

**Table S1.** The number of sequence entries in each data processing step.

| Input | Filtered | Denoised | Merged | Non-chimeric | | Contaminant filtered |
| --- | --- | --- | --- | --- | --- | --- |
| 4298014 | 3890235 | 3807848 | 3609859 | 3477692 | 1218950 | |

**Table S2.** The standard curve regression equations of four alkaloids.

| Alkaloids | Regression equation | R^2^ |
| --- | --- | --- |
| Protopine | y=4.3493x+24.816 | 0.9990 |
| Allocryptopine | y=3.5352x+7.9271 | 0.9993 |
| Sanguinarine | y=25.666x+111.18 | 0.9995 |
| Chelerythrine | y=32.352x-4.694 | 0.9992 |

**Table S3.** Spearman Correlation coefficients showing the relationships between endophytic bacterial genera and four alkaloids. *Significant differences (*P*<0.05) are indicated in bold.

| Phylum | Genus | Protopine | | Allocryptopine | | Sanguinarine | | Chelerythrine | |
| --- | --- | --- | --- | --- | --- | --- | --- | --- | --- |
|  |  | r | p | r | p | r | p | r | p |
| Thermi |  | 0.283 | 0.180 | -0.173 | 0.419 | 0.000 | 0.998 | -0.231 | 0.277 |
|  | B-42 | 0.045 | 0.834 | -0.105 | 0.624 | 0.227 | 0.286 | 0.226 | 0.288 |
|  | Deinococcus | 0.283 | 0.181 | -0.170 | 0.426 | 0.012 | 0.955 | -0.219 | 0.304 |
|  | Thermus | 0.123 | 0.566 | -0.030 | 0.891 | 0.080 | 0.711 | 0.047 | 0.826 |
|  | Truepera | 0.213 | 0.317 | 0.050 | 0.816 | 0.383 | 0.065 | 0.351 | 0.093 |
| Acidobacteria |  | -0.243 | 0.253 | 0.171 | 0.423 | -0.241 | 0.257 | -0.151 | 0.482 |
|  | Candidatus Koribacter | 0.105 | 0.624 | 0.256 | 0.227 | 0.030 | 0.888 | 0.106 | 0.624 |
|  | Candidatus Solibacter | 0.105 | 0.624 | 0.256 | 0.227 | 0.030 | 0.888 | 0.106 | 0.624 |
|  | Edaphobacter | 0.126 | 0.557 | 0.480 | **0.018** | 0.033 | 0.877 | 0.133 | 0.535 |
|  | Terriglobus | -0.256 | 0.227 | -0.256 | 0.227 | -0.288 | 0.173 | -0.302 | 0.152 |
| Actinobacteria |  | -0.036 | 0.867 | 0.337 | 0.108 | -0.531 | **0.008** | -0.424 | **0.039** |
|  | Actinoallomurus | -0.136 | 0.528 | -0.015 | 0.944 | 0.030 | 0.888 | 0.045 | 0.834 |
|  | Actinomyces | -0.256 | 0.227 | -0.256 | 0.227 | -0.288 | 0.173 | -0.302 | 0.152 |
|  | Agrococcus | -0.328 | 0.117 | -0.328 | 0.117 | -0.321 | 0.126 | -0.342 | 0.102 |
|  | Amycolatopsis | 0.100 | 0.642 | 0.319 | 0.129 | -0.123 | 0.567 | 0.010 | 0.964 |
|  | Arthrobacter | 0.045 | 0.834 | -0.105 | 0.624 | 0.227 | 0.286 | 0.226 | 0.288 |
|  | Brachybacterium | -0.023 | 0.914 | -0.190 | 0.374 | 0.461 | **0.023** | 0.429 | **0.036** |
|  | Brevibacterium | -0.015 | 0.944 | -0.045 | 0.834 | 0.348 | 0.096 | 0.347 | 0.097 |
|  | Cellulosimicrobium | -0.015 | 0.944 | -0.045 | 0.834 | 0.348 | 0.096 | 0.347 | 0.097 |
|  | Conexibacter | -0.092 | 0.670 | 0.228 | 0.283 | -0.030 | 0.891 | 0.128 | 0.551 |
|  | Corynebacterium | -0.151 | 0.481 | -0.192 | 0.369 | 0.407 | **0.048** | 0.412 | **0.046** |
|  | Curtobacterium | 0.444 | **0.030** | 0.004 | 0.985 | 0.228 | 0.284 | 0.047 | 0.828 |
|  | Dermacoccus | 0.196 | 0.359 | 0.045 | 0.834 | 0.288 | 0.173 | 0.256 | 0.227 |
|  | Dietzia | -0.452 | **0.027** | -0.317 | 0.131 | -0.338 | 0.107 | -0.320 | 0.128 |
|  | Friedmanniella | 0.384 | 0.064 | 0.224 | 0.293 | 0.347 | 0.097 | 0.234 | 0.271 |
|  | Glycomyces | 0.045 | 0.834 | -0.105 | 0.624 | 0.227 | 0.286 | 0.226 | 0.288 |
|  | Gordonia | -0.243 | 0.253 | -0.309 | 0.142 | -0.080 | 0.710 | -0.091 | 0.672 |
|  | Herbiconiux | 0.105 | 0.624 | 0.256 | 0.227 | 0.030 | 0.888 | 0.106 | 0.624 |
|  | Iamia | 0.226 | 0.288 | 0.015 | 0.944 | -0.197 | 0.357 | -0.241 | 0.256 |
|  | Janibacter | 0.135 | 0.530 | 0.017 | 0.937 | 0.607 | **0.002** | 0.610 | **0.002** |
|  | Kibdelosporangium | 0.296 | 0.160 | 0.325 | 0.122 | 0.130 | 0.545 | 0.101 | 0.638 |
|  | Kineococcus | 0.658 | **0.000** | 0.262 | 0.216 | 0.350 | 0.094 | 0.157 | 0.463 |
|  | Kribbella | 0.102 | 0.634 | 0.493 | **0.014** | -0.003 | 0.987 | 0.110 | 0.608 |
|  | Kutzneria | 0.034 | 0.876 | 0.467 | **0.022** | -0.032 | 0.882 | 0.164 | 0.445 |
|  | Kytococcus | -0.256 | 0.227 | -0.256 | 0.227 | -0.288 | 0.173 | -0.302 | 0.152 |
|  | Lactococcus | 0.045 | 0.834 | -0.105 | 0.624 | 0.227 | 0.286 | 0.226 | 0.288 |
|  | Microbacterium | 0.387 | 0.062 | 0.726 | **0.000** | 0.125 | 0.560 | 0.207 | 0.332 |
|  | Microbispora | 0.196 | 0.359 | 0.045 | 0.834 | 0.288 | 0.173 | 0.256 | 0.227 |
|  | Micrococcus | -0.455 | **0.026** | -0.197 | 0.356 | -0.313 | 0.137 | -0.130 | 0.545 |
|  | Mycobacterium | -0.070 | 0.745 | 0.405 | **0.050** | -0.128 | 0.550 | 0.025 | 0.908 |
|  | Nesterenkonia | 0.325 | 0.121 | 0.107 | 0.617 | 0.413 | **0.045** | 0.392 | 0.058 |
|  | Nocardia | 0.140 | 0.515 | 0.451 | **0.027** | 0.013 | 0.951 | 0.107 | 0.620 |
|  | Nocardioides | 0.196 | 0.359 | 0.045 | 0.834 | 0.288 | 0.173 | 0.256 | 0.227 |
|  | Patulibacter | -0.105 | 0.624 | -0.136 | 0.528 | 0.197 | 0.357 | 0.196 | 0.359 |
|  | Phycicoccus | 0.196 | 0.359 | 0.045 | 0.834 | 0.288 | 0.173 | 0.256 | 0.227 |
|  | Pilimelia | 0.123 | 0.567 | 0.473 | **0.020** | -0.120 | 0.576 | 0.011 | 0.959 |
|  | Propionibacterium | -0.487 | **0.016** | -0.628 | **0.001** | -0.067 | 0.755 | -0.058 | 0.788 |
|  | Propionicimonas | -0.015 | 0.944 | -0.045 | 0.834 | 0.348 | 0.096 | 0.347 | 0.097 |
|  | Pseudonocardia | 0.326 | 0.119 | 0.491 | **0.015** | 0.194 | 0.365 | 0.204 | 0.338 |
|  | Quadrisphaera | 0.151 | 0.482 | 0.105 | 0.624 | 0.106 | 0.622 | -0.060 | 0.780 |
|  | Renibacterium | -0.304 | 0.149 | -0.257 | 0.225 | -0.344 | 0.100 | -0.350 | 0.094 |
|  | Rhodococcus | -0.300 | 0.154 | -0.392 | 0.058 | 0.147 | 0.493 | 0.169 | 0.430 |
|  | Rubrobacter | -0.166 | 0.439 | -0.166 | 0.439 | 0.257 | 0.225 | 0.302 | 0.152 |
|  | Saccharopolyspora | -0.090 | 0.677 | -0.133 | 0.535 | 0.389 | 0.060 | 0.388 | 0.061 |
|  | Salinibacterium | -0.137 | 0.524 | 0.145 | 0.500 | -0.185 | 0.387 | -0.031 | 0.888 |
|  | Serinicoccus | -0.015 | 0.944 | -0.045 | 0.834 | 0.348 | 0.096 | 0.347 | 0.097 |
|  | Streptomyces | 0.344 | 0.100 | 0.496 | **0.014** | 0.117 | 0.586 | 0.238 | 0.262 |
|  | Unclassified | 0.024 | 0.912 | 0.314 | 0.135 | -0.428 | 0.037 | -0.283 | 0.180 |
|  | Varibaculum | -0.045 | 0.834 | 0.136 | 0.528 | 0.076 | 0.725 | 0.075 | 0.726 |
|  | Yonghaparkia | -0.166 | 0.439 | -0.166 | 0.439 | 0.257 | 0.225 | 0.302 | 0.152 |
| Armatimonadetes |  | 0.163 | 0.446 | 0.192 | 0.368 | 0.089 | 0.681 | 0.145 | 0.500 |
|  | Fimbriimonas | 0.216 | 0.311 | 0.209 | 0.327 | 0.005 | 0.981 | 0.060 | 0.782 |
| Bacteroidetes |  | 0.137 | 0.525 | 0.378 | 0.069 | -0.316 | 0.133 | -0.332 | 0.113 |
|  | Bacteroides | -0.615 | **0.001** | -0.617 | **0.001** | -0.512 | **0.011** | -0.542 | **0.006** |
|  | Capnocytophaga | -0.196 | 0.359 | -0.196 | 0.359 | -0.151 | 0.480 | -0.166 | 0.439 |
|  | Chryseobacterium | -0.054 | 0.803 | -0.176 | 0.411 | 0.176 | 0.409 | 0.125 | 0.562 |
|  | Cytophaga | 0.165 | 0.440 | 0.436 | **0.033** | -0.187 | 0.381 | -0.080 | 0.709 |
|  | Dyadobacter | -0.129 | 0.548 | 0.339 | 0.105 | -0.008 | 0.972 | 0.193 | 0.365 |
|  | Flavisolibacter | 0.187 | 0.382 | 0.122 | 0.569 | 0.377 | 0.069 | 0.376 | 0.070 |
|  | Flavobacterium | 0.202 | 0.344 | 0.624 | **0.001** | 0.101 | 0.638 | 0.301 | 0.153 |
|  | Hymenobacter | 0.527 | **0.008** | 0.088 | 0.684 | 0.269 | 0.204 | 0.081 | 0.707 |
|  | KSA1 | -0.075 | 0.726 | -0.075 | 0.727 | 0.318 | 0.130 | 0.302 | 0.152 |
|  | Larkinella | 0.158 | 0.462 | 0.274 | 0.195 | 0.164 | 0.444 | 0.287 | 0.174 |
|  | Niastella | 0.328 | 0.118 | 0.677 | **0.000** | -0.172 | 0.423 | 0.047 | 0.827 |
|  | Pedobacter | -0.312 | 0.138 | -0.005 | 0.982 | 0.109 | 0.613 | 0.247 | 0.245 |
|  | Porphyromonas | -0.015 | 0.944 | -0.045 | 0.834 | 0.348 | 0.096 | 0.347 | 0.097 |
|  | Prevotella | 0.024 | 0.913 | -0.111 | 0.607 | 0.412 | **0.046** | 0.410 | **0.047** |
|  | Prevotella | -0.003 | 0.989 | -0.056 | 0.795 | 0.105 | 0.626 | 0.095 | 0.660 |
|  | Rudanella | 0.316 | 0.132 | 0.196 | 0.359 | 0.136 | 0.526 | -0.060 | 0.780 |
|  | Saprospira | 0.151 | 0.482 | 0.105 | 0.624 | 0.106 | 0.622 | -0.060 | 0.780 |
|  | Sediminibacterium | 0.075 | 0.726 | 0.286 | 0.175 | -0.106 | 0.622 | 0.015 | 0.944 |
|  | Siphonobacter | -0.090 | 0.677 | -0.133 | 0.535 | 0.389 | 0.060 | 0.388 | 0.061 |
|  | Sphingobacterium | -0.344 | 0.100 | -0.245 | 0.248 | -0.093 | 0.665 | -0.079 | 0.715 |
|  | Spirosoma | 0.643 | **0.001** | 0.236 | 0.268 | 0.442 | **0.031** | 0.235 | 0.269 |
|  | Sporocytophaga | -0.226 | 0.288 | -0.286 | 0.175 | -0.288 | 0.173 | -0.302 | 0.152 |
|  | Terrimonas | -0.346 | 0.097 | -0.346 | 0.097 | -0.288 | 0.173 | -0.347 | 0.097 |
|  | Wautersiella | -0.015 | 0.944 | -0.045 | 0.834 | 0.348 | 0.096 | 0.347 | 0.097 |
| Chlamydiae |  | -0.01 | 0.962 | 0.293 | 0.165 | -0.281 | 0.184 | -0.266 | 0.209 |
|  | Candidatus Protochlamydia | 0.149 | 0.487 | 0.573 | **0.003** | -0.203 | 0.342 | 0.011 | 0.958 |
|  | Candidatus Rhabdochlamydia | 0.309 | 0.142 | 0.447 | **0.028** | 0.135 | 0.529 | 0.110 | 0.608 |
| Chloroflexi |  | 0.348 | 0.096 | 0.621 | **0.001** | 0.198 | 0.354 | 0.296 | 0.160 |
|  | Ardenscatena | -0.105 | 0.624 | -0.136 | 0.528 | 0.197 | 0.357 | 0.196 | 0.359 |
| Euryarchaeota |  | -0.095 | 0.658 | 0.051 | 0.814 | -0.187 | 0.381 | -0.114 | 0.595 |
|  | Halostagnicola | 0.075 | 0.726 | 0.286 | 0.175 | -0.106 | 0.622 | 0.015 | 0.944 |
| Firmicutes |  | -0.763 | **0.000** | -0.713 | 0.**000** | -0.370 | 0.075 | -0.267 | 0.207 |
|  | Abiotrophia | 0.286 | 0.175 | 0.226 | 0.288 | 0.166 | 0.437 | 0.166 | 0.439 |
|  | Aerococcus | -0.136 | 0.528 | -0.015 | 0.944 | 0.030 | 0.888 | 0.045 | 0.834 |
|  | Anaerococcus | -0.417 | **0.043** | -0.459 | **0.024** | -0.415 | **0.044** | -0.470 | **0.021** |
|  | Aneurinibacillus | 0.105 | 0.624 | 0.256 | 0.227 | 0.030 | 0.888 | 0.106 | 0.624 |
|  | Anoxybacillus | -0.396 | 0.055 | -0.396 | 0.055 | -0.321 | 0.126 | -0.376 | 0.070 |
|  | Bacillus | -0.257 | 0.226 | 0.081 | 0.705 | -0.469 | **0.021** | -0.270 | 0.202 |
|  | Blautia | -0.166 | 0.439 | -0.166 | 0.439 | 0.257 | 0.225 | 0.302 | 0.152 |
|  | Caldicoprobacter | -0.201 | 0.346 | -0.121 | 0.572 | -0.164 | 0.444 | -0.175 | 0.414 |
|  | Catonella | 0.286 | 0.175 | 0.226 | 0.288 | 0.166 | 0.437 | 0.166 | 0.439 |
|  | Clostridium | -0.084 | 0.695 | -0.128 | 0.552 | 0.398 | 0.054 | 0.397 | 0.055 |
|  | Coprococcus | -0.271 | 0.200 | -0.292 | 0.166 | 0.025 | 0.909 | -0.021 | 0.923 |
|  | Dialister | -0.105 | 0.624 | -0.136 | 0.528 | 0.197 | 0.357 | 0.196 | 0.359 |
|  | Enterococcus | -0.226 | 0.288 | -0.286 | 0.175 | -0.288 | 0.173 | -0.302 | 0.152 |
|  | Exiguobacterium | -0.136 | 0.528 | -0.015 | 0.944 | 0.030 | 0.888 | 0.045 | 0.834 |
|  | Faecalibacterium | -0.256 | 0.227 | -0.256 | 0.227 | -0.288 | 0.173 | -0.302 | 0.152 |
|  | Gemella | 0.286 | 0.175 | 0.226 | 0.288 | 0.166 | 0.437 | 0.166 | 0.439 |
|  | GW-34 | -0.015 | 0.944 | -0.045 | 0.834 | 0.348 | 0.096 | 0.347 | 0.097 |
|  | Lactobacillus | 0.417 | **0.043** | 0.061 | 0.778 | 0.248 | 0.243 | 0.144 | 0.502 |
|  | Lysinibacillus | -0.196 | 0.359 | -0.196 | 0.359 | -0.151 | 0.480 | -0.166 | 0.439 |
|  | Megasphaera | -0.256 | 0.227 | -0.256 | 0.227 | -0.288 | 0.173 | -0.302 | 0.152 |
|  | Moryella | 0.286 | 0.175 | 0.226 | 0.288 | 0.166 | 0.437 | 0.166 | 0.439 |
|  | Natronobacillus | -0.015 | 0.944 | -0.045 | 0.834 | 0.348 | 0.096 | 0.347 | 0.097 |
|  | Oceanobacillus | -0.116 | 0.589 | -0.220 | 0.301 | 0.043 | 0.841 | 0.032 | 0.883 |
|  | Paenibacillus | -0.103 | 0.632 | 0.083 | 0.699 | 0.236 | 0.268 | 0.338 | 0.106 |
|  | Parvimonas | 0.286 | 0.175 | 0.226 | 0.288 | 0.166 | 0.437 | 0.166 | 0.439 |
|  | Peptoniphilus | 0.105 | 0.624 | 0.256 | 0.227 | 0.030 | 0.888 | 0.106 | 0.624 |
|  | Ruminococcus | -0.015 | 0.944 | -0.045 | 0.834 | 0.348 | 0.096 | 0.347 | 0.097 |
|  | Saccharibacillus | 0.045 | 0.834 | -0.105 | 0.624 | 0.227 | 0.286 | 0.226 | 0.288 |
|  | Salinicoccus | -0.196 | 0.359 | -0.196 | 0.359 | -0.151 | 0.480 | -0.166 | 0.439 |
|  | Sporanaerobacter | -0.351 | 0.093 | -0.306 | 0.147 | -0.321 | 0.126 | -0.296 | 0.160 |
|  | Sporosarcina | -0.243 | 0.253 | -0.309 | 0.142 | -0.080 | 0.710 | -0.091 | 0.672 |
|  | Staphylococcus | -0.728 | **0.000** | -0.701 | **0.000** | -0.328 | 0.118 | -0.270 | 0.203 |
|  | Streptococcus | -0.190 | 0.374 | -0.261 | 0.218 | 0.064 | 0.766 | 0.041 | 0.851 |
|  | Tepidimicrobium | -0.057 | 0.791 | -0.161 | 0.451 | -0.017 | 0.936 | -0.050 | 0.818 |
|  | Thermoanaerobacterium | -0.316 | 0.132 | -0.316 | 0.132 | -0.288 | 0.173 | -0.196 | 0.359 |
|  | Tissierella_Soehngenia | -0.196 | 0.359 | -0.196 | 0.359 | -0.151 | 0.480 | -0.166 | 0.439 |
|  | Veillonella | 0.286 | 0.175 | 0.226 | 0.288 | 0.166 | 0.437 | 0.166 | 0.439 |
|  | Virgibacillus | 0.103 | 0.632 | 0.112 | 0.603 | 0.335 | 0.109 | 0.342 | 0.102 |
|  | Weissella | -0.180 | 0.399 | -0.247 | 0.245 | 0.025 | 0.909 | 0.013 | 0.951 |
| Fusobacteria |  | 0.219 | 0.303 | 0.263 | 0.214 | 0.126 | 0.557 | 0.144 | 0.502 |
|  | Cetobacterium | 0.151 | 0.482 | 0.316 | 0.132 | -0.151 | 0.480 | -0.121 | 0.575 |
|  | Fusobacterium | 0.286 | 0.175 | 0.226 | 0.288 | 0.166 | 0.437 | 0.166 | 0.439 |
|  | Leptotrichia | -0.105 | 0.624 | -0.136 | 0.528 | 0.197 | 0.357 | 0.196 | 0.359 |
| Nitrospirae |  | 0.105 | 0.624 | 0.256 | 0.227 | 0.03 | 0.888 | 0.106 | 0.624 |
|  | Nitrospira | 0.105 | 0.624 | 0.256 | 0.227 | 0.030 | 0.888 | 0.106 | 0.624 |
| Planctomycetes |  | -0.111 | 0.605 | 0.049 | 0.820 | -0.321 | 0.126 | -0.265 | 0.211 |
|  | Planctomyces | 0.151 | 0.482 | 0.316 | 0.132 | -0.151 | 0.480 | -0.121 | 0.575 |
| Proteobacteria |  | 0.051 | 0.813 | -0.225 | 0.289 | 0.608 | **0.002** | 0.577 | **0.003** |
|  | Achromobacter | -0.196 | 0.359 | -0.196 | 0.359 | -0.151 | 0.480 | -0.166 | 0.439 |
|  | Acidovorax | -0.250 | 0.239 | -0.050 | 0.817 | -0.091 | 0.672 | 0.052 | 0.811 |
|  | Acinetobacter | -0.867 | **0.000** | -0.707 | **0.000** | -0.395 | 0.056 | -0.305 | 0.147 |
|  | Afifella | -0.226 | 0.288 | -0.286 | 0.175 | -0.288 | 0.173 | -0.302 | 0.152 |
|  | Afipia | 0.087 | 0.687 | 0.614 | **0.001** | -0.115 | 0.592 | 0.096 | 0.654 |
|  | Agrobacterium | -0.035 | 0.872 | 0.261 | 0.218 | -0.103 | 0.633 | 0.030 | 0.891 |
|  | Anaeromyxobacter | -0.015 | 0.944 | -0.045 | 0.834 | 0.348 | 0.096 | 0.347 | 0.097 |
|  | Antarctobacter | -0.015 | 0.944 | -0.045 | 0.834 | 0.348 | 0.096 | 0.347 | 0.097 |
|  | Aquicella | -0.098 | 0.649 | 0.169 | 0.430 | -0.189 | 0.377 | -0.099 | 0.644 |
|  | Arcobacter | 0.226 | 0.288 | 0.015 | 0.944 | -0.197 | 0.357 | -0.241 | 0.256 |
|  | Asticcacaulis | 0.065 | 0.763 | 0.369 | 0.076 | -0.144 | 0.503 | -0.038 | 0.861 |
|  | Azohydromonas | -0.177 | 0.408 | -0.177 | 0.409 | 0.414 | **0.045** | 0.436 | **0.033** |
|  | Azomonas | 0.151 | 0.482 | 0.316 | 0.132 | -0.151 | 0.480 | -0.121 | 0.575 |
|  | Balneimonas | -0.015 | 0.944 | -0.045 | 0.834 | 0.348 | 0.096 | 0.347 | 0.097 |
|  | Bdellovibrio | 0.279 | 0.187 | 0.452 | **0.027** | -0.244 | 0.250 | -0.204 | 0.340 |
|  | Bosea | 0.039 | 0.856 | 0.317 | 0.131 | 0.203 | 0.340 | 0.320 | 0.127 |
|  | Bradyrhizobium | -0.388 | 0.061 | 0.151 | 0.480 | -0.455 | **0.026** | -0.220 | 0.303 |
|  | Brevundimonas | -0.102 | 0.634 | -0.207 | 0.333 | -0.017 | 0.936 | 0.030 | 0.890 |
|  | Burkholderia | -0.083 | 0.701 | 0.352 | 0.091 | -0.126 | 0.557 | 0.043 | 0.841 |
|  | Caulobacter | -0.253 | 0.232 | 0.038 | 0.860 | -0.096 | 0.656 | 0.015 | 0.946 |
|  | Cellvibrio | 0.075 | 0.726 | 0.286 | 0.175 | -0.106 | 0.622 | 0.015 | 0.944 |
|  | Chitinophaga | 0.266 | 0.209 | 0.700 | **0.000** | 0.029 | 0.891 | 0.170 | 0.427 |
|  | Collimonas | 0.015 | 0.944 | 0.346 | 0.097 | -0.076 | 0.725 | 0.136 | 0.527 |
|  | Comamonas | -0.105 | 0.624 | -0.136 | 0.528 | 0.197 | 0.357 | 0.196 | 0.359 |
|  | Corallococcus | 0.151 | 0.482 | 0.105 | 0.624 | 0.106 | 0.622 | -0.060 | 0.780 |
|  | Cystobacter | 0.426 | **0.038** | 0.068 | 0.752 | 0.406 | **0.049** | 0.244 | 0.251 |
|  | Dechloromonas | 0.105 | 0.624 | 0.256 | 0.227 | 0.030 | 0.888 | 0.106 | 0.624 |
|  | Delftia | -0.089 | 0.681 | 0.275 | 0.193 | 0.245 | 0.249 | 0.382 | **0.066** |
|  | Devosia | 0.014 | 0.948 | 0.506 | **0.012** | -0.178 | 0.404 | 0.009 | 0.968 |
|  | Dokdonella | 0.102 | 0.635 | 0.641 | **0.001** | -0.117 | 0.585 | 0.097 | 0.652 |
|  | Elstera | -0.372 | 0.074 | -0.368 | 0.077 | -0.415 | **0.044** | -0.390 | 0.059 |
|  | Enhydrobacter | -0.856 | **0.000** | -0.824 | **0.000** | -0.324 | 0.123 | -0.233 | 0.273 |
|  | Ensifer | 0.105 | 0.624 | 0.256 | 0.227 | 0.030 | 0.888 | 0.106 | 0.624 |
|  | Enterobacter | -0.226 | 0.288 | -0.286 | 0.175 | -0.288 | 0.173 | -0.302 | 0.152 |
|  | Erwinia | -0.227 | 0.285 | -0.334 | 0.111 | 0.231 | 0.277 | 0.235 | 0.269 |
|  | Gluconacetobacter | -0.769 | **0.000** | -0.715 | **0.000** | -0.397 | 0.055 | -0.397 | 0.055 |
|  | Halomonas | 0.196 | 0.359 | 0.045 | 0.834 | 0.288 | 0.173 | 0.256 | 0.227 |
|  | Herbaspirillum | -0.660 | **0.000** | -0.448 | **0.028** | -0.751 | **0.000** | -0.675 | **0.000** |
|  | Hydrocarboniphaga | -0.070 | 0.746 | 0.300 | 0.154 | 0.185 | 0.386 | 0.320 | 0.128 |
|  | Hylemonella | -0.095 | 0.658 | 0.179 | 0.404 | -0.211 | 0.322 | -0.058 | 0.787 |
|  | Hyphomicrobium | -0.230 | 0.279 | 0.220 | 0.302 | -0.318 | 0.129 | -0.116 | 0.589 |
|  | Inquilinus | 0.073 | 0.733 | 0.518 | **0.010** | -0.172 | 0.421 | 0.033 | 0.880 |
|  | Janthinobacterium | 0.564 | **0.004** | 0.169 | 0.429 | 0.495 | **0.014** | 0.379 | 0.068 |
|  | Kaistobacter | 0.228 | 0.283 | 0.392 | 0.058 | 0.212 | 0.320 | 0.423 | **0.040** |
|  | Klebsiella | -0.126 | 0.558 | -0.257 | 0.225 | 0.023 | 0.914 | 0.012 | 0.955 |
|  | Labrys | 0.084 | 0.697 | 0.537 | **0.007** | -0.029 | 0.892 | 0.176 | 0.410 |
|  | Legionella | -0.041 | 0.851 | 0.415 | **0.044** | 0.123 | 0.567 | 0.312 | 0.138 |
|  | Leptothrix | -0.136 | 0.528 | -0.015 | 0.944 | 0.030 | 0.888 | 0.045 | 0.834 |
|  | Limnohabitans | -0.286 | 0.175 | -0.226 | 0.288 | -0.288 | 0.173 | -0.241 | 0.256 |
|  | Luteibacter | -0.050 | 0.817 | 0.187 | 0.382 | -0.051 | 0.815 | 0.045 | 0.837 |
|  | Luteimonas | 0.015 | 0.944 | 0.346 | 0.097 | -0.076 | 0.725 | 0.136 | 0.527 |
|  | Lysobacter | -0.683 | **0.000** | -0.670 | **0.000** | -0.332 | 0.113 | -0.352 | 0.092 |
|  | Mesorhizobium | -0.133 | 0.535 | 0.083 | 0.702 | 0.075 | 0.727 | 0.086 | 0.689 |
|  | Methylibium | -0.726 | **0.000** | -0.525 | **0.008** | -0.760 | **0.000** | -0.649 | **0.001** |
|  | Methylobacterium | 0.263 | 0.215 | -0.203 | 0.339 | -0.121 | 0.572 | -0.316 | 0.132 |
|  | Methylotenera | 0.105 | 0.624 | 0.256 | 0.227 | 0.030 | 0.888 | 0.106 | 0.624 |
|  | Methylovirgula | -0.136 | 0.528 | -0.015 | 0.944 | 0.030 | 0.888 | 0.045 | 0.834 |
|  | Morganella | 0.105 | 0.624 | 0.256 | 0.227 | 0.030 | 0.888 | 0.106 | 0.624 |
|  | Mycoplana | -0.372 | 0.074 | -0.368 | 0.077 | -0.415 | **0.044** | -0.390 | 0.059 |
|  | Neisseria | 0.286 | 0.175 | 0.226 | 0.288 | 0.166 | 0.437 | 0.166 | 0.439 |
|  | Nevskia | 0.165 | 0.440 | 0.436 | **0.033** | -0.187 | 0.381 | -0.080 | 0.709 |
|  | Nitrobacter | -0.136 | 0.528 | -0.015 | 0.944 | 0.030 | 0.888 | 0.045 | 0.834 |
|  | Novosphingobium | -0.128 | 0.551 | 0.159 | 0.459 | 0.088 | 0.682 | 0.231 | 0.276 |
|  | Pantoea | -0.053 | 0.805 | -0.303 | 0.151 | 0.373 | 0.073 | 0.366 | 0.079 |
|  | Paracoccus | -0.262 | 0.216 | -0.222 | 0.298 | -0.023 | 0.916 | 0.050 | 0.816 |
|  | Pedomicrobium | 0.105 | 0.624 | 0.256 | 0.227 | 0.030 | 0.888 | 0.106 | 0.624 |
|  | Pelomonas | -0.781 | **0.000** | -0.691 | **0.000** | -0.553 | **0.005** | -0.509 | **0.011** |
|  | Perlucidibaca | 0.075 | 0.726 | 0.286 | 0.175 | -0.106 | 0.622 | 0.015 | 0.944 |
|  | Phenylobacterium | -0.038 | 0.859 | 0.423 | **0.040** | 0.106 | 0.623 | 0.302 | 0.151 |
|  | Phyllobacterium | -0.145 | 0.499 | 0.329 | 0.117 | -0.093 | 0.666 | 0.098 | 0.648 |
|  | Pigmentiphaga | -0.166 | 0.439 | -0.166 | 0.439 | 0.257 | 0.225 | 0.302 | 0.152 |
|  | Polaromonas | -0.070 | 0.744 | 0.244 | 0.250 | -0.122 | 0.570 | 0.014 | 0.947 |
|  | Procabacter | 0.151 | 0.482 | 0.105 | 0.624 | 0.106 | 0.622 | -0.060 | 0.780 |
|  | Pseudochrobactrum | -0.256 | 0.227 | -0.256 | 0.227 | -0.288 | 0.173 | -0.302 | 0.152 |
|  | Pseudomonas | -0.369 | 0.076 | -0.363 | 0.082 | 0.356 | 0.087 | 0.447 | **0.029** |
|  | Pseudoxanthomonas | -0.019 | 0.931 | 0.412 | **0.045** | 0.024 | 0.911 | 0.200 | 0.349 |
|  | Ralstonia | -0.815 | **0.000** | -0.775 | **0.000** | -0.318 | 0.130 | -0.264 | 0.213 |
|  | Rheinheimera | -0.434 | **0.034** | -0.389 | 0.060 | -0.415 | **0.044** | -0.317 | 0.131 |
|  | Rhizobium | -0.132 | 0.539 | 0.364 | 0.080 | -0.173 | 0.419 | 0.056 | 0.797 |
|  | Rhodanobacter | -0.251 | 0.238 | 0.297 | 0.158 | -0.127 | 0.553 | 0.111 | 0.606 |
|  | Rhodobacter | -0.067 | 0.755 | -0.088 | 0.683 | 0.480 | **0.018** | 0.467 | **0.021** |
|  | Rhodoplanes | 0.077 | 0.721 | 0.522 | **0.009** | -0.073 | 0.733 | 0.052 | 0.809 |
|  | Rickettsia | 0.363 | 0.082 | 0.290 | 0.169 | 0.243 | 0.254 | 0.046 | 0.830 |
|  | Roseomonas | 0.283 | 0.180 | 0.118 | 0.583 | 0.190 | 0.374 | 0.144 | 0.502 |
|  | Rubellimicrobium | -0.017 | 0.936 | -0.123 | 0.566 | 0.642 | **0.001** | 0.639 | **0.001** |
|  | Serratia | -0.392 | 0.058 | -0.429 | **0.036** | -0.310 | 0.141 | -0.296 | 0.160 |
|  | Shewanella | -0.058 | 0.789 | -0.143 | 0.504 | 0.643 | **0.001** | 0.645 | **0.001** |
|  | Shinella | 0.015 | 0.944 | 0.346 | 0.097 | -0.076 | 0.725 | 0.136 | 0.527 |
|  | Skermanella | -0.166 | 0.439 | -0.166 | 0.439 | 0.257 | 0.225 | 0.302 | 0.152 |
|  | Sorangium | 0.075 | 0.726 | 0.286 | 0.175 | -0.106 | 0.622 | 0.015 | 0.944 |
|  | Sphingobium | 0.106 | 0.620 | 0.421 | **0.041** | 0.439 | **0.032** | 0.530 | **0.008** |
|  | Sphingomonas | 0.257 | 0.226 | -0.196 | 0.358 | 0.554 | **0.005** | 0.428 | **0.037** |
|  | Sphingopyxis | 0.081 | 0.707 | 0.540 | **0.007** | -0.028 | 0.897 | 0.182 | 0.396 |
|  | Stenotrophomonas | -0.067 | 0.757 | -0.172 | 0.421 | 0.313 | 0.137 | 0.341 | 0.103 |
|  | Steroidobacter | 0.075 | 0.729 | 0.540 | **0.007** | -0.021 | 0.923 | 0.189 | 0.377 |
|  | Telmatospirillum | -0.155 | 0.470 | 0.089 | 0.678 | -0.153 | 0.477 | -0.025 | 0.907 |
|  | Thermomonas | -0.196 | 0.359 | -0.196 | 0.359 | -0.151 | 0.480 | -0.166 | 0.439 |
|  | Uliginosibacterium | 0.075 | 0.726 | 0.286 | 0.175 | -0.106 | 0.622 | 0.015 | 0.944 |
|  | Variovorax | 0.105 | 0.626 | 0.552 | **0.005** | -0.031 | 0.886 | 0.098 | 0.650 |
|  | Zoogloea | -0.196 | 0.359 | -0.196 | 0.359 | -0.151 | 0.480 | -0.166 | 0.439 |
| Tenericutes |  | -0.481 | **0.017** | -0.544 | **0.007** | -0.685 | **0.000** | -0.659 | **0.000** |
|  | Acholeplasma | -0.346 | 0.097 | -0.346 | 0.097 | -0.288 | 0.173 | -0.347 | 0.097 |
|  | Candidatus Phytoplasma | -0.478 | **0.018** | -0.541 | **0.007** | -0.685 | **0.000** | -0.653 | **0.001** |
| Verrucomicrobia |  | -0.035 | 0.871 | 0.296 | 0.16 | -0.251 | 0.237 | -0.09 | 0.675 |
|  | Chthoniobacter | 0.082 | 0.703 | 0.332 | 0.113 | -0.062 | 0.775 | -0.039 | 0.858 |
